# Temperature-fastened sodium inactivation accounts for energy efficient cortical action potentials in mammalian brains

**DOI:** 10.1186/1471-2202-13-S1-P5

**Published:** 2012-07-16

**Authors:** Yuguo Yu

**Affiliations:** 1 Center for Computational Systems Biology, School of Life Sciences, Fudan University, Shanghai, 200433, China

## 

Recent experimental evidencs showed that action potential (AP) generation in mammalian, versus invertebrate, axons is remarkably energy efficient [1]. Here we perform both computational (based on both traditional Hodgkin-Huxley model [2] and a cortical axon model [3] whose parameters are modified from experimental data) and experimental studies. Each supports that temperature is a major factor which directly modulates the level of energy cost of APs. Temperature increase results in a remarkable decrease in time constant of sodium channel closing and an increase in inactivation level of Na+ channel due to the Q10 effect (which quantifies the temperature dependent rate of biochemical reactions). This results in a marked reduction in overlap of the inward Na+, and outward K+ currents. As a consequence, the Na+ entry ratio gradually reaches to 1 (the theoretical optimal level, which requires only minimal Na+ charge for generating an AP) as temperature rises. Moreover, we also notice a remarkable exponential increase in firing rate and an exponential decrease in spike duration by both experimental and model studies. The total energy charge in response to a signal reaches a global minimum when temperature is around 37-42 ^o^C. This suggests that warm body temperatures may help the mammalian brain to operate with minimal energy cost.

In addition, classic investigations by Hodgkin of squid giant axon revealed an excess entry of approximately 4 times as much Na^+^ as minimally required to generate an AP [4]. This value of 4 times excess Na^+^ entry has figured prominently in estimates of the distribution of the sources of energy consumption in the mammalian brain. Here we have to point out that this calculation is based on original Hodgkin-Huxley model with a temperature at 18 ^o^C. It should not be used for calculation of mammalian brain energy budget since mammalian animals have a warm body temperature around 37 ^o^C, around which the sodium entry ratio is close to 1.3.

**Figure 1:**
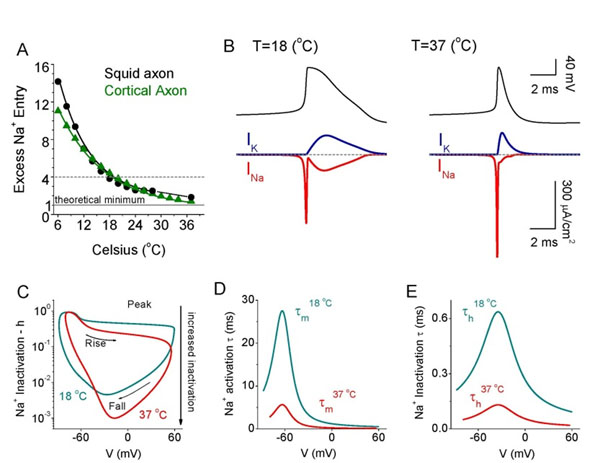
Energy efficiency of AP increases as temperature increases. A. Sodium entry ratio (SER, defined as ∫I_Na_(t)dt/(C_m_∆V), where I_Na_(t) is Na+ current, C_m_ is capacitance, ∆V is the change in voltage during an AP) vs temperature. For both squid axon and cortical axon models, increasing temperature strongly decreases SER during AP. At 18^o^ C, SER is approximately 4, while at 37^o^ C, SER reaches 1.89 and 1.41. B. Cortical AP, I_Na_ and I_K_ at 18 and 37^o^ C, respectively. C. The inactivation level of Na^+^ channel increases as temperature increases, indicating a more closed state for a high temperature, decreasing the leaky Na+ through membrane. D. Decrease of Na+ activation time constant as temperature increases. E. Decrease of Na+ inactivation time constant as temperature increases.
